# Role of Tonsillectomy in the Management of Carcinomas of Unknown Primary of the Head and Neck: A Retrospective Study Based on p16 Analysis

**DOI:** 10.3389/fonc.2020.594168

**Published:** 2020-10-21

**Authors:** Pauline Podeur, Julien Mancini, Jean Delgrande, Laure Santini, Sébastien Salas, Stéphanie Wong, Antoine Giovanni, Patrick Dessi, Justin Michel, Thomas Radulesco, Nicolas Fakhry

**Affiliations:** ^1^Department of Otorhinolaryngology, Head and Neck Surgery, Centre Hospitalier Universitaire la Conception, Assistance Publique–Hôpitaux de Marseille, Aix Marseille Univ, Marseille, France; ^2^Aix-Marseille Univ, INSERM, IRD, APHM, UMR1252, SESSTIM, Department of Public Health (BIOSTIC), Hôpital de la Timone, Marseille, France; ^3^Medical Oncology Department, Centre Hospitalier (CHU) La Timone, Marseille, France; ^4^Radiation Oncology Department, Hôpital Timone Adultes, Marseille, France

**Keywords:** unknown primary, oropharyngeal cancer, human papillomavirus, head and neck cancer, squamous cell carcinoma

## Abstract

**Purpose:**

To evaluate the impact of tonsillectomy on the detection of the primary tumor, based on p16 immunohistochemistry analysis, in patients with cervical unknown primary of squamous cell carcinoma (SCC-CUP).

**Methods:**

This was a retrospective study of 63 patients, included from January 2008 to December 2017 in a single institution. All patients had an initial assessment with physical examination, CT scan of the neck and chest, whole body FDG-PET CT, and endoscopy under general anesthesia, which failed to determine the primary tumor.

**Results:**

Forty-seven out of the 63 patients had an ipsi- or bilateral tonsillectomy which revealed 12 tonsil cancers (26%). The tonsil primary was ipsilateral to positive nodes in 10 cases, contralateral in 1 case and, in 1 case, the patient had bilateral neck involvement. The analysis of the p16 status was carried out in 41/63 patients (65%). Among the 32 patients who had a p16 analysis and tonsillectomy, the rate of primary detection was 59% (10/17) for p16-postives and 0% (0/15) for p16-negatives (p < 0.001).

**Conclusion:**

These results suggest that an extended work-up should be systematically proposed including bilateral tonsillectomy (+/- mucosectomy of the base of tongue) in SCC-CUP p16-positive patients but not in p16-negatives.

## Purpose

Carcinomas of unknown primary (CUP) of the head and neck are lymph node metastases with no primary tumor identified after a work-up including a physical examination and imaging tests (CT scan of the neck and chest, whole body FDG-PET CT). When no primary cancer is found, endoscopy under general anesthesia is performed, possibly associated with ipsi- or bilateral tonsillectomy.

Squamous cell carcinoma (SCC) is the most common histology of head and neck CUP. The incidence of head and neck CUP of squamous cell carcinoma (SCC-CUP) is rare, accounting for 1–4% of all head and neck cancers, and mortality is high, with mean 5-year survival rates that vary widely depending on the study, ranging from 24 to 79% of cases (1, 2). The detection of a primary tumor is an important goal to help improve both overall and disease-free survival. It is likely that this is related to the potential adaptation of treatment to the primary tumor by proposing a targeted treatment with curative surgery and a decrease in morbidity of an “over-treatment” (saving on adjuvant radiotherapy or modified radiation fields) (3).

It would appear that HPV or EBV status could point to a primary oropharyngeal or nasopharyngeal tumor. SCC-CUP represents a diagnostic challenge and, to date, there is no consensus on whether tonsillectomy should be performed as a single or bilateral procedure and on whether base of tongue mucosectomy should be conducted. Moreover, few studies have evaluated the impact of HPV status on the rate of discovery of the primary tumor (3, 4).

The aim of this study was to evaluate the impact of tonsillectomy on identification of the primary tumor, based on p16 immunohistochemistry analysis, in patients with SCC-CUP.

## Methods

### Ethical Considerations

All procedures performed in studies involving human participants were in accordance with the ethical standards of the institutional and/or national research committee and with the 1964 Helsinki declaration and its later amendments or comparable ethical standards. Informed consent was obtained from all participants included in the study. Authorization to conduct this study was obtained from the Ethical Committee of our institution (Assistance Publique des Hôpitaux de Marseille, no. 2018-28).

### Study Design

This was a retrospective, monocentric study analyzing the records of 63 patients managed from January 2008 to December 2017 with SCC-CUP (histologically confirmed by lymph node sampling) with no identified primary tumor after clinical examination, standardized imaging (CT scan of the neck and chest and whole body FDG-PET CT) and endoscopy under general anesthesia. Tonsillectomies were performed either during the initial endoscopy when no suspicious area was discovered or, in case of directed biopsy of any suspicious areas, during a second procedure when biopsies were finally negatives. Patients for whom a primary was visible during the endoscopy were not included in the study. Concerning the lymph node sampling, 33 patients (52%) had an adenectomy or lymph node open biopsy performed at another center and were then referred to our institution. Thirty-one patients (48%) had an initial Fine-needle aspiration cytology with, in all cases, confirmation of diagnosis (frozen section + histological analysis) after lymph node dissection. Patients with a history of head and neck cancer or radiotherapy of the neck were not included. Mean age of the patients was 63 years (range, 38 to 84). There were 51 males and 12 females. The rate of discovering a primary tonsillar tumor was noted as well as patient characteristics (sex, age, alcohol and tobacco consumption, lymph node location, TNM stage, histological criteria of aggressiveness, and HPV and EBV status).

### Immunohistochemical Analysis

The presence of HPV in tumor cells was based on overexpression of the p16 protein using immunohistochemistry. The secondary antibody was clone E6H4 reference 6695248001, from the Roche laboratory, using Ventana BenchMark ULTRA automaton. The presence of EBV was tested using *in situ* hybridization with the EBER VENTANA-ROCHE probe (Epstein Barr virus Early RNA Probe; REF: 800.2842; GTIN: 04015630971923) using Ventana BenchMark ULTRA automaton.

Testing for the presence of EBV and HPV was performed in 41/63 patients (65%). These analyses had been performed at the time of initial assessment for 6 patients and were performed retrospectively for 35 patients. For 22 patients, the EBV or p16 status could not be determined as histological samples were not available.

### Statistical Analysis

Categorical data were compared using Fisher’s exact tests. Non-parametric Mann-Whitney tests were used to compare ordinal data. P values of less than 0.05 were taken to be statistically significant. All statistical analyses were two-sided and performed using IBM SPSS Statistics 20.0 (IBM Inc., New York, USA).

## Results

In the whole series, 12 primary tonsillar tumors were found (19%). Tonsillectomy was performed in 47 patients (bilateral in 36 and unilateral in 11) representing 75% of the patients in the series. Deep biopsies, without tonsillectomy, were performed in 10 patients (16%). Six other patients (9%) who had a history of bilateral tonsillectomy in childhood with no residual tonsil visible on endoscopy, had biopsies of the tonsil fossa. Three of the 47 patients with tonsillectomy (6%) had a postoperative hemorrhagic complication requiring hemostasis under general anesthesia: in two cases, the bleeding occurred in the tonsillar fossa ipsilateral to the lymphadenopathy. Among these two cases, we observed a primary carcinoma in one case. In one case, there was contralateral bleeding with no tumor.

For the 47 patients who had tonsillectomy, the procedure revealed 12 tonsil cancers (26%). The tonsil primary was ipsilateral to positive nodes in 10 cases (84%) and contralateral in 1 case (8%), and in 1 case (8%), the patient had bilateral neck involvement.

All the primary tumors were found on the tonsillectomy specimens. No primary tumors were found after deep biopsies without tonsillectomy. The median size of the primary tumors found after tonsillectomy was 6 mm (range, 2 to 18 mm). Among the 36 patients who had bilateral tonsillectomy, one primary contralateral to lymphadenopathy was found (3%).

For the 47 patients who had tonsillectomy, the procedure revealed 12 tonsil cancers (26%). The tonsil primary was ipsilateral to positive nodes in 10 cases (84%) and contralateral in 1 case (8%), and in 1 case (8%), the patient had bilateral neck involvement.

No statistically significant differences were found for age, sex, alcohol consumption, M stage, location of lymphadenopathies, extracapsular spread, and tumor differentiation (**Table 1**).

**Table 1:** Comparison of patients with or without primary finding.

	Tonsil primary		No primary		Overall population	p (Fisher test)
	N	%	N	%	N (%)	
**Population**	12	19%	51	81%	63	
**Sex**						
Male	10	83%	41	80%	51 (81%)	0.99
Female	2	17%	10	20%	12 (19%)	
**Tobacco consumption**						
Yes	7	58%	44	86%	51 (81%)	**0.03**
No	5	42%	6	12%	11 (17%)	
Not available	0		1	2%	1 (2%)	
**Alcohol consumption**						
Yes	6	50%	27	53%	33 (52%)	0.75
No	6	50%	21	41%	27 (43%)	
Not available	0		3	6%	3 (5%)	
**Stage N**						
N1	7	58%	16	31%	23 (36%)	0.04*
N2	4	33%	16	31%	20 (32%)	
N3	1	8%	19	37%	20 (32%)	
**Stage M**						
M0	12	100%	50	98%	62 (98%)	0.99
M1	0		1	2%	1 (2%)	
**Tonsillectomy**						
Unilateral	5	42%	6	12%	11 (17.5%)	0.17
Bilateral	7	58%	29	57%	36 (57%)	
No tonsillectomy	0	0%	16	31%	16 (25.5%)	
**Lymph node levels involved**						
I	1	8%	8	16%	9 (14%)	0.99
II	11	92%	42	82%	53 (84%)	0.67
III	3	25%	21	41%	24 (38%)	0.35
IV	1	8%	10	20%	11 (17%)	0.45
V	2	17%	8	16%	10 (16%)	0.99
**Bilateral lymph node involvement**						
Yes	1	8%	4	8%	5 (8%)	0.99
No	11	92%	47	92%	58 (92%)	
**Extracapsular spread**						
Yes	2	17%	19	37%	21 (34%)	0.30
No	9	75%	29	57%	38 (60%)	
Not available	1	8%	3	6%	4 (6%)	
**p16 status**						
Positive	10	83%	8	16%	18 (29%)	**<0.001**
Negative	0		23	45%	23 (36%)	
Not available	2	17%	20	39%	22 (35%)	
**Tumor differentiation**						
Well differentiated	6	50%	22	43%	28 (44%)	0.58*
Moderate differentiation	2	17%	7	14%	9 (14%)	
Poor/undifferentiated	4	33%	19	37%	23 (37%)	
Not available	0		3	6%	3 (5%)	

*Mann-Whitney test for ordinal data.

Bold characters denote statistical significance.

Of the 41 patients for which immunohistochemical analysis was performed, none was positive for EBV. Eighteen out of 41 patients (44%) were p16-positive, among which a primary tonsil tumor was found in 10 cases (56%). Twenty-three out of 41 patients (56%) were p16-negative, among which no primary tonsil tumor was found. Lastly, a primary tonsillar tumor was found in two patients whose p16 status was unknown.

Of the 32 patients who had a p16 analysis (all EBV-negative) and an ipsi- or bilateral tonsillectomy, 17 were p16-positive and 15 were p16-negative. Ten primary tonsil tumors were found, all in p16-positive patients. The primary detection rate in p16-positive patients with tonsillectomy was therefore 10/17 (59%) versus 0% for p16-negative patients (p < 0.001).

## Discussion

### Benefits of Tonsillectomy

In our study, a tonsil primary tumor was found in 19% of cases (12 patients out of 63). However, this percentage is probably underestimated because 16 patients had only tonsil deep biopsies and no tonsillectomy.

In a cohort of 126 patients with SCC-CUP, Waltonen et al. reported a positive yield in 30% of patients who underwent tonsillectomy. In comparison, in the same study, deep biopsies identified the malignancy in only 3% of cases, reflecting the fact that some tumors are small and located within the tonsillar crypts and therefore cannot be identified by biopsy alone (5).

In our study, among the patients undergoing tonsillectomy, the latter was bilateral in 77% of cases and unilateral in 23% of cases. This distribution is similar to that found in 2016 by Farnebo et al. studying the management of patients with SCC-CUP in 22 main centers in five Nordic countries (Iceland, Norway, Sweden, Finland, and Denmark). Routine bilateral tonsillectomy was performed in about 80% of cases compared to 20% for unilateral tonsillectomy (6).

In our series, out of the 47 patients undergoing tonsillectomy, a primary tumor was found in 26% of cases, with a primary contralateral to the lymphadenopathy in 3% of the cases who underwent bilateral tonsillectomy. Our results are consistent with the literature reporting that ipsilateral tonsillectomy has a detection rate of 18 to 45%. However, they are lower for contralateral tonsillectomy with a likely detection rate ranging from 10 to 25% (7).

Di Maio et al. performed a systematic review and meta-analysis to evaluate the effectiveness of palatine tonsillectomy in patients with SCC-CUP. They analyzed 14 studies comprising 673 patients who underwent 416 palatine tonsillectomies, 338 performed during examination under anesthesia, and 78 managed with transoral robotic surgery (TORS). A total of 140 occult tonsillar malignancies were identified. Of these, 124 (89%) were ipsilateral, 2 were (1%) contralateral, and 14 were (10%) synchronous bilateral. A meta-analysis of 11 out of the 14 studies showed an overall detection by tonsillectomy rate of 0.34 (99% confidence interval, 0.23–0.46). The authors concluded that palatine tonsillectomy is a valuable diagnostic tool and that bilateral tonsillectomy should be considered mainly not only because of the non-negligible number of bilateral/contralateral occult tonsillar tumors reported in the literature but also because out of 204 bilateral tonsillectomies performed (from a total of 416), 2 bleeding episodes were reported in only one of the included articles (8). In our series, bleeding occurred in one (3%) contralateral tonsil fossa among the 36 patients undergoing bilateral tonsillectomy.

The American Society of Clinical Oncology has recently published evidence-based recommendations on the diagnosis and management of squamous cell carcinoma of unknown primary in the head and neck. They recommend that patients should undergo a complete operative upper aerodigestive tract evaluation of mucosal sites at risk (oral cavity, nasopharynx, oropharynx, hypopharynx, and larynx), including directed biopsy of any suspicious areas. Random biopsies of nonsuspicious areas have a low yield and should not be performed. For patients with unilateral lymphadenopathy, if a primary site is not confirmed on initial evaluation, then the surgeon should perform ipsilateral palatine tonsillectomy. If palatine tonsillectomy fails to identify a primary, then ipsilateral lingual tonsillectomy may be performed. Bilateral palatine tonsillectomy may be considered according to clinical suspicion, at the discretion of the surgeon. For patients with bilateral lymphadenopathy, if a primary site is not confirmed on endoscopic examination, then the surgeon may perform unilateral lingual tonsillectomy on the side with the greater nodal burden and may perform contralateral lingual tonsillectomy if the ipsilateral procedure fails to identify a primary. Bilateral palatine tonsillectomy after bilateral lingual tonsillectomy should be avoided (9).

However, there is no international consensus on whether tonsillectomy should be a single or bilateral procedure and whether it should be combined with an ipsi- or bilateral base of tongue mucosectomy depending on p16 status (3, 10).

### Impact of p16 Immunohistochemistry Analysis

It is widely accepted that the base of the tongue and the tonsils are the most common primary tumor sites found in the work-up for SCC-CUP (1, 3, 4).

The question is whether p16 status influences the detection rate of the primary tumor in the oropharynx. In our study, 10 of the 12 carcinomas found in the tonsils were p16-positive (83%), while for the other 2 patients, the p16 status was unknown. Also, among patients in whom a tonsillar primary was found, there were statistically fewer smokers than among those without a detected primary. In addition, the former had a lower lymph node stage. Most importantly, no tonsillar primary was found in p16-negative patients. Finally, among the patients in our series who underwent tonsillectomy, the detection rate of a tonsillar primary in p16-positive patients was 53% compared to 0% in p16-negative patients.

The role of p16 status on the rate of primary tumor detection in the oropharynx has been very little studied in the literature.

In the systematic review by Di Maio et al. the p16 status was available for 116 out of 673 patients. Of these, 104 (90%) were p16-positive and 12 (10%) p16-negative, but no information is given about the rate of primary findings in these patients (8). Ryan et al. analyzed 80 p16-positive patients with SCC-CUP. After direct laryngoscopy with biopsies, 29/80 (35%) primary tumors were identified. Thirty-four patients with negative biopsies underwent palatine tonsillectomy. Fifteen of these 34 (44%) revealed the primary tumor, yielding a cumulative identification of 44/80 (55%) (11).

In our study, the presence of HPV in tumor cells was based on overexpression of the p16 protein using immunohistochemistry. According to the guideline the College of American Pathologists, the preferred method for initial high risk-HPV testing of tissue specimens (core biopsy or excisions) in high-prevalence settings is p16 immunohistochemistry, which is a sensitive surrogate marker (12).

### Decision-Making Based on p16 Status

Our results showed that oropharyngeal primaries were found exclusively in p16-positive patients and never in p16-negative patients. The detection rate of a tonsil primary in p16-positive patients undergoing tonsillectomy was 53% in our series. This result is probably underestimated since not all our patients had bilateral tonsillectomy and none of them underwent base of tongue mucosectomy.

These findings highlight the need to intensify the search for the primary at oropharynx level in p16-positive patients. It is necessary, therefore, to ascertain the p16 status as early as possible by means of a cervical lymph node sample. In this way, p16-positive patients, in which there is the greatest likelihood of finding a primary tumor, could benefit from maximum sampling of the oropharynx. These patients could thus benefit from bilateral tonsillectomy possibly associated, at the same time or at a second stage, with a base of tongue mucosectomy to optimize the search for the primary tumor, as suggested by several authors (4, 13). Mehta et al. evaluated 10 patients with unknown primary tumors of the head and neck. All patients underwent a cervical biopsy, positron-emission tomography/computed tomography, formal endoscopy, and bilateral tonsillectomy. When the initial endoscopy and biopsies failed to locate a primary tumor, all patients underwent transoral robotic base of tongue resection. A primary was found in 9/10 (90%) patients, of which 8 out of the 9 (89%) were HPV-positive (13).

On the other hand, tonsillectomy and/or mucosectomy of the base of the tongue for p16-negative patients is much more debatable since, in our series, no primary was found in p16-negative patients who underwent tonsillectomy. This observation was already made by Kubic et al. (14), who analyzed the rate of primary detection in 23 p16-negative patients using TORS base of tongue mucosectomy. The primary tumor was identified in only 3 out of 23 cases (13%). In these three cases, the tumor was found in the ipsilateral base-of-tongue specimen in contrast with their previous series showing a tumor identification rate of 80% in the HPV-positive patients (4, 14).

## Conclusion

Early determination of p16 status from a lymph node sample is important as it allows preferential referral to a primary oropharyngeal tumor and boosts the search for the primary tumor (bilateral tonsillectomy +/- base of tongue mucosectomy) in p16-positive patients. On the other hand, tonsillectomy and base of tongue mucosectomy for p16-negative patients are much more debatable, since, in our series, no primary was found in these patients.

## Data Availability Statement

The raw data supporting the conclusions of this article will be made available by the authors, without undue reservation.

## Ethics Statement

The studies involving human participants were reviewed and approved by: Authorization to conduct this study was obtained from the Ethical Committee of our institution (Assistance Publique des Hôpitaux de Marseille, n° 2018-28). The patients/participants provided their written informed consent to participate in this study.

## Author Contributions

Study concepts: NF and LS. Study design: NF and SW. Data acquisition: PP, JD, and LS. Quality control of data and algorithms: JMa. Data analysis and interpretation: PP and SS. Statistical analysis: JMa. Manuscript preparation: NF, PP, and TR. Manuscript editing: AG, PD, and JMi. Manuscript review: PD and NF. All authors contributed to the article and approved the submitted version.

## Conflict of Interest

The authors declare that the research was conducted in the absence of any commercial or financial relationships that could be construed as a potential conflict of interest.
